# Phenotypic switch of smooth muscle cells in paediatric chronic intestinal pseudo‐obstruction syndrome

**DOI:** 10.1111/jcmm.16367

**Published:** 2021-03-03

**Authors:** Delphine Martire, Sarah Garnier, Sébastien Sagnol, Annick Bourret, Stéphane Marchal, Norbert Chauvet, Amandine Guérin, Dominique Forgues, Dominique Berrebi, Christophe Chardot, Marc Bellaiche, John Rendu, Nicolas Kalfa, Sandrine Faure, Pascal de Santa Barbara

**Affiliations:** ^1^ PhyMedExp Université de Montpellier CNRS INSERM Montpellier France; ^2^ Visceral Paediatric Surgery Unit CHU de Montpellier Université de Montpellier Montpellier France; ^3^ Department of Paediatric Gastroenterology Assistance Publique Hôpitaux (APHP) Hospital Robert Debré Paris France; ^4^ Paediatric Surgery Necker‐Enfants malades Hospital Paris France; ^5^ Centre Hospitalier Universitaire de Grenoble Alpes Biochimie Génétique et Moléculaire Grenoble France; ^6^ Present address: Beta Innov SAS Montpellier France

**Keywords:** chronic intestinal pseudo‐obstruction Disease, intestinal motility disorders, PDGFR pathway, plasticity, smooth muscle cells

## Abstract

Smooth Muscle Cells (SMC) are unique amongst all muscle cells in their capacity to modulate their phenotype. Indeed, SMCs do not terminally differentiate but instead harbour a remarkable capacity to dedifferentiate, switching between a quiescent contractile state and a highly proliferative and migratory phenotype, a quality often associated to SMC dysfunction. However, phenotypic plasticity remains poorly examined in the field of gastroenterology in particular in pathologies in which gut motor activity is impaired. Here, we assessed SMC status in biopsies of infants with chronic intestinal pseudo‐obstruction (CIPO) syndrome, a life‐threatening intestinal motility disorder. We showed that CIPO‐SMCs harbour a decreased level of contractile markers. This phenotype is accompanied by an increase in Platelet‐Derived Growth Factor Receptor‐alpha (PDGFRA) expression. We showed that this modulation occurs without origin‐related differences in CIPO circular and longitudinal‐derived SMCs. As we characterized PDGFRA as a marker of digestive mesenchymal progenitors during embryogenesis, our results suggest a phenotypic switch of the CIPO‐SMC towards an undifferentiated stage. The development of CIPO‐SMC culture and the characterization of SMC phenotypic switch should enable us to design therapeutic approaches to promote SMC differentiation in CIPO.

## 1 INTRODUCTION

A distinguishing feature of the smooth muscle lineage is the capacity of all smooth muscle cells (SMCs) from vasculature, airways, gastrointestinal (GI) tract and urogenital tract to reversibly modulate their phenotype, switching between contractile and proliferative phenotypes.^1^, ^2^, ^3^ While SMC phenotypic plasticity is extensively studied in vascular diseases, very few studies have been reported in the field of gastroenterology, in particular in pathologies characterized by GI motility disorders.^2^, ^3^, ^4^

Chronic intestinal pseudo‐obstruction (CIPO) is the most life‐threatening intestinal motility disorder and comprises a heterogeneous group of conditions that affect the function of intestinal neuro‐musculature components.^5^, ^6^ Diagnostic and therapeutic approaches are variable and morbidity remains high. The pathophysiology is poorly understood. Paediatric CIPO is characterized by the chronic inability of the GI tract to propel its contents and mimics mechanical obstruction in the absence of any organic obstruction occluding the intestine.^7^ Several case reports suggest a role of immunomodulation in children with specific inflammatory neuropathies and/or myopathies.^8^, ^9^ Most cases of CIPO are sporadic, although X‐linked, autosomal dominant and recessive forms have been identified.^10^ Smooth muscle lesion, classified as digestive myopathies, has been described, in adult and childhood patients with CIPO.^5^, ^6^, ^11^, ^12^, ^13^ More recently, CIPO has been associated with mutations in genes that are expressed and/or involved in smooth muscle function, such as Filamin A (*FLNA*),^10^ Actin χ^2^ (*ACTG2*),^14^ Shugoshin‐like 1 (*SGOL1*),^15^ Myosin Heavy chain *11* (*MYH11*) ^16^ and Leimodin *1* (*LMOD1*).^17^ *ACTG2*, which encodes gamma enteric smooth muscle actin, was found to be mutated in almost 40% of children with CIPO.^18^ However, contradictory studies questioned the predictive value of the alteration of the smooth muscle in CIPO patients ^19^ and muscular examination has not been systematically analysed in paediatric patients with CIPO, mainly due to the poor knowledge on the molecular mechanisms involved in digestive smooth muscle cell (SMC) homeostasis.^2^

Digestive SMCs originate from the splanchnopleural mesoderm that differentiates along the radial axis, giving rise to the smooth muscle and submucosal layers.^20^ During development, mesenchymal progenitors first enter into a determination programme which is characterized by the expression of MYOCARDIN, the Serum Response Factor (SRF) co‐activator that defines the SMC lineage and induces the early expression of alpha Smooth Muscle Actin (αSMA).^21^ Then, progenitors start to elongate, cluster and express proteins involved in smooth muscle contractility, such as Calponin.^2^ These processes require the regulated activity of several downstream signalling pathways, such as the Bone Morphogenetic Protein (BMP), Fibroblast Growth Factor (FGF), NOTCH and HIPPO pathways.^22^, ^23^, ^24^, ^25^, ^26^ SMC dedifferentiation is induced by the reactivation of developmental processes through modulation of the signalling pathways such as BMP and/or FGF.^24^, ^27^, ^28^

In this study, we examined the differentiation and maturity status of SMCs from paediatric patients with CIPO. To this purpose, we set up a cellular model for CIPO. Using this model, we bring evidences that SMC of CIPO patients harbour a decreased expression of contractile markers. This phenotype is accompanied by an increased expression of Platelet‐Derived Growth Factor (PDGF) receptor‐alpha (PDGFRA) and consequently an activation of ERK signalling pathway. We found similar results in muscular tissues from CIPO patients. Our data strongly suggest that a phenotypic switch occurs in the SMCs of Paediatric CIPO patients.

## 2 MATERIALS AND METHODS

### 2.1 Isolation of SMC from CIPO tissues

Full‐thickness intestinal biopsy specimens were obtained from nine paediatric patients who met the clinical criteria for CIPO (5:4 male to female ratio; from 6 to 16 years of age) (CIPO samples). Control specimens (CTL) were from the normal part of the intestine of paediatric patients with Hirschsprung's disease selected after histological characterization (Figure S1). The use of human tissues was approved by the local Ethics Committee (Comité de Protection des Personnes Sud‐Méditerranée IV, N°DC‐2012‐1600). Paraffin‐embedded sections (8 µm thick) were immunostained using standard procedures.^29^

To derive SMC cultures from smooth muscle fibres, we carefully dissected smooth muscle fibres from paediatric CIPO and CTL muscle strips to avoid submucosa, vascular and myenteric plexus contamination. Intestinal muscle cells were isolated from the intestinal muscle layers (CIPO1 to CIPO8) or from the circular (CIPO1ci and CIPO9ci) and longitudinal (CIPO1lg and CIPO9lg) muscle layers, using previously described techniques.^30^ Muscle cells isolated by enzymatic digestion (collagenase‐IA and soybean trypsin inhibitor [Sigma‐Aldrich]) were plated and expanded on collagen I‐coated plates (Corning® BioCoat™; VWR) with DMEM containing 200 µg/mL gentamycin, 200 U/mL penicillin, 200 µg/mL streptomycin, 2.5 µg/mL amphotericin B and 10% Foetal Bovine Serum (FBS). Epithelial cells, endothelial cells, neurons and interstitial cells of Cajal were not detected in the cultures using such as protocol ^31^ (data not shown).

### 2.2 Immunofluorescence analysis and flow cytometry

Paraffin‐embedded block was sectioned at 8 µm thick. After deparaffinization and antigen retrieval (in 0.01 M citrate buffer pH 6.0, at 96°C for 30 minutes), slides were immunostained using standard procedures.^29^ Tissue sections were incubated at room temperature with a rabbit anti‐PDGFRA antibody (1:200 dilution; Cell Signaling Technologies, #3164) and a mouse anti‐αSMA (1:200 dilution; Santa Cruz Biotechnologies, clone 1A4) or with a rabbit anti‐αSMA antibody (1:100 dilution; Abcam, #ab5694) and a mouse anti‐TUJ1 (1:500 dilution; Covance, #MMS‐435P) for 2 hours. After washing, sections were incubated with Alexa 555‐conjugated anti‐mouse and Alexa 488‐conjugated anti‐rabbit (1:2000 dilution; Invitrogen for both) antibodies for 30 minutes. Nuclei were stained with Hoechst (Molecular Probes). Sections were rinsed and mounted in Mounting medium (DAKO). Images were acquired using a Carl‐Zeiss AxioImager microscope. The fluorescence signal from each channel was captured sequentially to avoid cross‐talk between channels. Immunofluorescence control experiments were performed without primary antibody, and no signal was detected (data not shown).

For cultures, SMCs were plated on collagen I‐coated coverslips (25,000 cells/cm^2^) in DMEM containing 10% FBS, or DMEM with 0.2% BSA and 5 μg/mL insulin to induce differentiation for 3 to 6 days.^24^ Mouse anti‐αSMA (1:200 dilution; Santa Cruz Biotechnologies, clone 1A4), rabbit anti‐αSMA (1:200 dilution; Abcam, #ab5694), goat anti‐MYOCARDIN (1:200 dilution; Santa Cruz Biotechnologies, clone M16), rabbit anti‐KI67 (1:200 dilution; Invitrogen, clone SP6) and rabbit anti‐PDGFRA (1:200 dilution; Cell Signaling Technologies, #3164) antibodies were used. Secondary anti‐mouse, anti‐rabbit or anti‐goat IgG coupled to Alexa 488 or 555 (1:1000 dilution; Life Technologies) was used. Nuclei were stained with Hoechst (1:500 dilution; Molecular Probes). Images were acquired using a Carl‐Zeiss AxioImager microscope. The number of MYOCARDIN‐, PDGFRA‐, KI67‐ and αSMA‐positive cells was quantified with the Fiji image analysis software. Image background was removed with a ‘rolling ball’ algorithm; a median filter was applied followed by thresholding. Images were converted to a binary format and quantified (number of positive cells relative to all nuclei). Positive cells were counted using the ‘analyse particle’ plugin. CIPO, CTL2 and CTL1 data were compared with the two‐tailed Mann‐Whitney test and the GraphPad Prism 6.0 software; n represents the number of biological samples. Each value used for statistical analyses was the mean value of three independent cultures at early passage (2‐3). For each experiment, we count from 200 to 500 cells for each condition. Results were considered significant when *P* <.05 (*), *P* <.01 (**), *P* <.001 (***) or *P* <.0001 (****).

For flow cytometry analysis, cells were incubated with Accutase (Sigma, #A6964) for 5 minutes at 37° to obtain a single cell suspension, and then centrifuged at 200 rcf for 10 minutes. Cells were re‐suspended in Wash buffer with phycoerythrin (PE) alone or with (PE)‐conjugated mouse anti‐CD140a antibodies (1:50, BD Pharmingen, # 556 002), and incubated on ice for 30 minutes, gently swirling every 10 minutes. Then, cells were washed and directly sorted by flow cytometry on a CANTO II FACS (Becton Dickinson) controlled through FACSDiva 8.0.1 software.

### 2.3 Protein extraction, PDGFRA stimulation and Western blotting

Cells were lysed in lysis buffer (20 mM Tris pH8, 50 mM NaCl, 1% NP40, complete^TM^ EDTA‐free Protease Inhibitor Cocktail (Roche), 2 mM activated orthovanadate (Sigma‐Aldrich) and PhosStop [Roche]). For PDGFRA stimulation, SMCs (25,000 cells/cm^2^) were plated on collagen I‐coated dishes in DMEM containing 10% FBS. After 24 hours, cells were rinsed twice with PBS and incubated in OPTIMEM medium without serum for 1 hour, followed by incubation with 10 ng/ml or 100 ng/ml recombinant human PDGF‐AA (Peprotech) at 37°C for 15 minutes. Cells were scraped in PBS/2 mM activated sodium orthovanadate and proteins were then extracted as before and analysed by Western blots.^32^ Protein concentration was determined using the RC DCTM Protein Assay kit (BioRad). Diluted protein samples were boiled in SDS‐PAGE buffer, separated by SDS‐PAGE in 12% acrylamide/BisAcrylamide gels and transferred to nitrocellulose membranes at 100 V for 1.5 hours. Membranes were incubated according to the Odyssey technology protocol (LI‐COR Biosystems) with mouse anti‐αSMA (1:500 dilution; Santa Cruz Biotechnologies, clone 1A4), rabbit anti‐PDGFRA (1:400 dilution; Cell Signaling Technologies, #3164), mouse anti‐GAPDH (1:10 000 dilution; Sigma‐Aldrich), mouse anti‐p44/42 MAPK (Erk1/Erk2) (1:400 dilution; Cell Signaling Technologies, L34F12, #4696) and rabbit anti‐Phospho‐p44/42 (Erk1/Erk2) (Thr202/Tyr2014) (1:400 dilution; Cell Signaling Technologies, 20G11, #4696) antibodies. Immunoblots were quantified using infrared‐labelled secondary antibodies and the Odyssey infrared imaging system (LI‐COR Biosystems).

### 2.4 Reverse transcription and quantitative polymerase chain reaction (RT‐qPCR)

Total RNA was extracted from CIPO and CTL intestinal muscle fibres or from SMC cultures with the HighPure RNA Isolation Kit (Roche). Total RNA from smooth muscle layers was extracted with TRIzol.^33^ RT was performed with the Verso cDNA Synthesis Kit (Thermo Scientific), and qPCR using the LightCycler technology (Roche Diagnostics). PCR primers (Table S1) were designed using the LightCycler Probe Design software‐2.0. Each sample was analysed in three independent experiments done in triplicate. Expression levels were determined with the LightCycler analysis software (version 3.5), relative to standard curves. Data were represented as the mean level of gene expression relative to the mean expression of the reference genes *GAPDH* and *RPLPO*. The relative mRNA expression was calculated using the 2^−ΔΔCT^ method. CIPO, CTL2 and CTL1 expression data were analysed with two‐tailed Mann‐Whitney tests and the GraphPad Prism 6.0 software; n represents the number of biological replicates. Each value used for statistical analyses was the mean value of three technical replicates. Results were considered significant when *P* < .05 (*), *P* < .01 (**), *P* < .001 (***) or *P* < .0001 (****).

### 2.5 *ACTG2* gene sequencing

Mutation screening was performed after amplification of the entire coding sequence of the *ACTG2* gene using patients’ DNA and a 3500XL Genetic Analyzer (ThermoFisher Scientific, Waltham, USA). Patient CIPO1 harbours a heterozygous missense variant (c.588G >C or p. Glu196Asp) that was not reported in the literature. Analysis of this variant deleteriousness with the Combined Annotation‐Dependent Depletion (CADD) online tool to predict the functional effects of human missense variants gave a score of 23.7.^34^ This analysis suggested that this variant is a putative damaging variant in a highly conserved region in the vertebrate orthologous and paralogous isoforms. Moreover, it was not reported in the Exome Sequence Variant database (http://evs.gs.washington.edu/EVS/) or the Exome Aggregation Consortium (ExAC) database. This variant was reported once in the ClinVar database as probably pathogenic and was classified as probably pathogenic according to the American College of Medical Genetics and Genomic and the Association for Molecular Pathology (ACMG‐AMP) guidelines.^35^

### 2.6 Whole‐mount in situ hybridization in chick gut

Timed fertilized white Leghorn eggs (Morizeau EARL) were incubated at 38°C in a humidified incubator (Coudelou) until used experimentally. Gastrointestinal (GI) tissues were dissected and staged by embryonic day (E).^36^ Dissected GI tissues were fixed in 4% paraformaldehyde for 1 h at room temperature, washed in PBS, gradually dehydrated in methanol in order to store the samples at −20°C for at least one night. Whole‐mount in situ hybridization analyses were carried out as described using antisense *TAGLN* ^25^ and *PDGFRA* ^37^ riboprobes. For whole‐mount in situ hybridization experiments, tissues were gradually rehydrated in PBS, washed in PBT (PBS, 0.1% Tween) and incubated for 1 hours in 6% hydrogen peroxide (Sigma, France). Samples were next permeabilized by treatment with proteinase K (10 μg/ml) for 10 minutes, washed with glycine in PBT and fixed in 4% paraformaldehyde/0.2% gluteraldehyde in PBT for 20 minutes. Tissues were then hybridized with antisense *PDGFRA* or *TAGLN* digoxigenin‐labelled (Roche) riboprobes overnight at 70°C. After post‐hybridization washes at 70°C, tissues were incubated in 10% sheep serum for 2.5 hours at room temperature and finally mixed with preabsorbed anti‐digoxigenin coupled with alkaline phosphatase antibody (Roche) overnight at 4°C. The complexes were detected with BM Purple, a chromogenic substrate for alkaline phosphatase (Roche). Images were acquired using a Nikon Multizoom AZ100 stereomicroscope.

## 3 RESULTS

### 3.1 Smooth muscle cells of CIPO patients harbour decreased level of contractile markers

**FIGURE 1:**
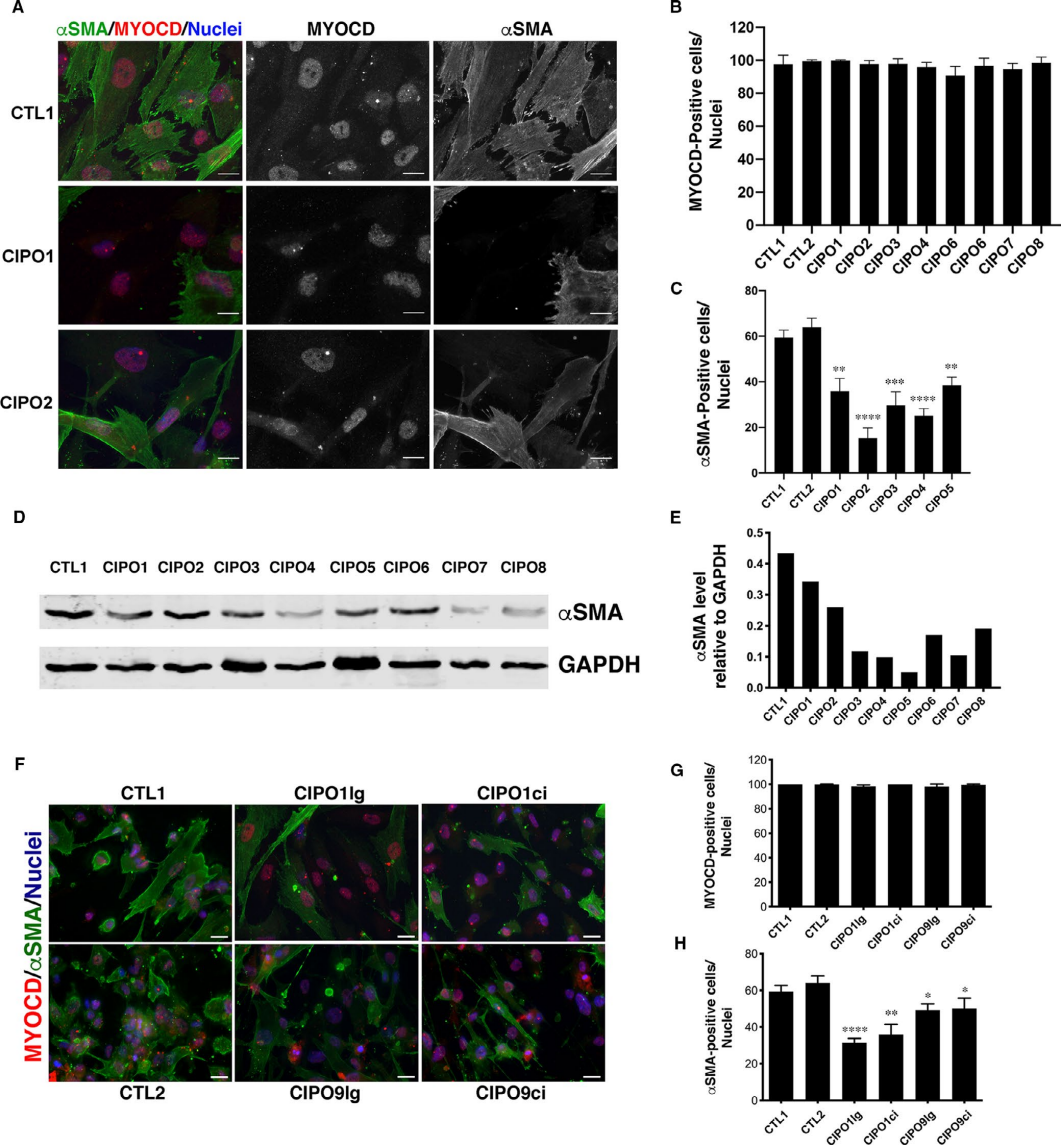
Development of CIPO‐SMC cultures and identification of SMC alteration. (A) Representative immunofluorescence images of CIPO‐ and CTL‐SMC cultures incubated with anti‐αSMA (green) and anti‐MYOCARDIN (MYOCD, red) antibodies. Nuclei were visualized with Hoechst (blue). Scale bar = 20 µm. Percentage of MYOCARDIN‐positive (B) and αSMA‐positive cells (C) in CIPO‐ and CTL‐SMC cultures relative to the total number of nuclei (Hoechst staining). Values are presented as the mean ± SD of n = 5 experiments at early passages (2‐3). CTL1 and CIPO data were compared with the two‐tailed Mann‐Whitney test. (D) Representative Western blot analysis of αSMA expression in CIPO‐ and CTL‐SMC extracts. (E) Quantification relative to GAPDH expression of the Western blot results shown in D. (F) Representative immunofluorescence analysis of SMC cultures from circular and longitudinal smooth muscle layers with anti‐αSMA (green) and anti‐MYOCARDIN (MYOCD, red) antibodies. Nuclei were visualized with Hoechst (blue). Scale bar = 40 µm. Percentage of MYOCARDIN‐positive (G) and αSMA‐positive cells (H) in the indicated SMC cultures relative to the total number of cells

To examine the differentiated status of smooth muscle cells of CIPO patients, we set up a cellular model of paediatric CIPO syndrome. As control, we used SMC cells isolated from the ganglionic zone of Hirschsprung patient. In control tissues, αSMA expression was found localized homogenously both in the circular and longitudinal smooth muscle layers, supporting that ganglionic zone of HRSC patients harbours normal SMC differentiation status (Figure S1). Importantly, CIPO1 patient carries a heterozygous mutation in the *ACTG2* gene (c.588G >C; p. E196D) (Figure S2). Primary SMC culture purity was assessed by immunofluorescence using MYOCARDIN, the SRF‐co‐activator which controls numerous and early steps of SMC differentiation ^21^ (Figure 1A). We found that 90.65% to 99.77% of cells in both CIPO‐ and Control‐SMC cultures are positive for MYOCARDIN expression (Figure 1B). In all conditions, MYOCDARDIN staining harbours a nuclear localization (Figure 1A). When analysing the expression of αSMA (early smooth muscle marker) in MYOCARDIN‐positive cells (Figure 1A), we found that the number of αSMA‐expressing cells was lower in CIPO‐SMC cultures (15.33% to 38.42%) compared to Control conditions (59.4% and 63.97%) (Figure 1C). αSMA protein level was analysed by Western blot on extracts performed on CIPO and CTL cultures (Figure 1D; Figure S3A). Western blot quantifications revealed a reduced level of αSMA protein in all CIPO cultures (n = 8/8) examined compared to Controls (Figure 1E; Figure S3B).

As digestive musculature is organized in circular (inner) and longitudinal (outer) muscle layers that contribute both to peristalsis, we next examined whether the decreased expression of contractile markers was observed in both muscle layers. For this, we isolated SMCs from the circular (CIPO1ci and CIPO9ci) and longitudinal (CIPO1lg and CIPO9lg) muscle layers. MYOCARDIN was detected in all cultures (98.25% to 100% of cells) (Figure 1F,G). We found that the number of αSMA‐expressing cells was lower in CIPO‐SMC cultures derived respectively from the circular and longitudinal layers (32.68% to 51.61%) compared to Control conditions (65.37% and 60.21%) (Figure 1H). All together, these data show that the number of αSMA‐positive cells is lower in CIPO‐SMC cultures than in control cultures without origin‐related differences.

### 3.2 PDGFRA expression defines gastrointestinal mesenchymal progenitors

**FIGURE 2:**
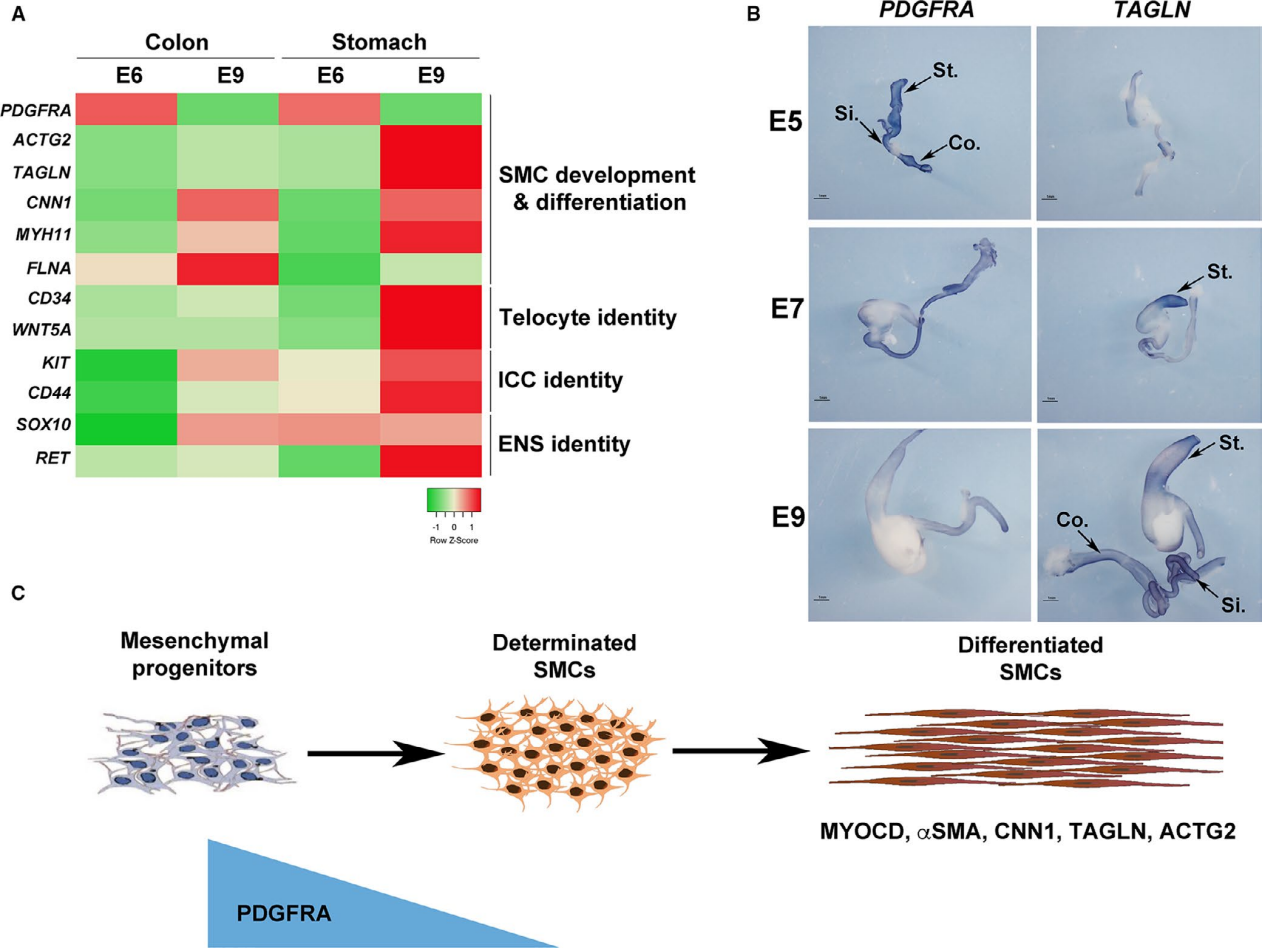
Spatial and temporal expression of *PDGFRA* mRNA during embryonic development of the chick GI tract. (A) Heatmap for *PDGFRA* in chick colon and stomach (E6 and E9) samples. The highest signals are in res, and lowest signals are in green. *PDGFRA* mRNA is strongly expressed in the early stages, whereas SMC differentiation markers (*ACTG2*, *TAGLN*, *CNN1*, *MYH11*, *FLNA*) are strongly expressed at E9. *CD34* and *WNT5A* are specific from telocytes. *KIT* and *CD44* are associated to ICCs. *SOX10* and *RET* characterize ENS identity. (B) Whole‐mount in situ hybridization analysis of *PDGFRA* and *TAGLN* in the chick E5, E7 and E9 GI tract. *PDGFRA* is strongly expressed in the GI mesenchyme at E5 whereas *TAGLN* is strongly present at E9. (C) Schematic representation of the expression of PDGFRA during GI mesenchymal progenitors to differentiated SMCs

Unlike mature skeletal and cardiac myocytes, SMC retain the developmental potential to modulate their cellular phenotype from a differentiated stage to a more immature state, with the decreased expression of contractile markers and the reactivation of developmental signalling pathways. As previously hypothesized,^2^, ^3^ we postulated that such a mechanism could be involved in the impairment of gut motility observed in CIPO patients. To identify genes that define the SMC immaturity, we screened for genes that demonstrated higher level of expression during the process of SMC differentiation using the chick embryo model. We compared profiles of genes expressed in 6‐day‐old (E6), a stage in which mesenchymal progenitors are not yet differentiated in SMCs (progenitor stage), to those expressed at E9, a stage in which SMCs are differentiated (differentiated stage) ^23^, ^24^, ^25^, ^38^ and found Platelet‐Derived Growth Factor (PDGF) receptor‐alpha (PDGFRA) to be highly expressed in the mesenchymal progenitors (Figure 2A). Its profile contrasts to the ones of *SOX10* and *RET* (Enteric Nervous System (ENS) markers),^23^, ^38^ *KIT* and *CD44* (Interstitial Cells of Cajal (ICC) markers),^39^, ^40^ and *CD34* and *WNT5A* (telocyte markers) ^41^, ^42^ and differentiated smooth muscle markers (*ACTG2*, *CNN1*, *FLNA*, *MYH11* and *TAGLN*) that harbour highly expression at differentiated stage (Figure 2A). The PDGF pathway includes two receptors (PDGFRA and B) and four ligands. PDGFRs are involved in the development and differentiation of many organs ^43^ and PDGFRs play key survival factors for vascular SMCs, and are the principal regulators of vascular SMC phenotype.^44^ To confirm our transcriptomic approach, we performed in situ hybridization analyses on chick GI tracts and found *PDGFRA* strongly expressed in the whole GI mesenchyme at E5 (Figure 2B). *PDGFRA* expression decreases from E7 onwards and its expression is barely detectable at differentiated stage (Figure 2B) in contrast to the one of *TAGLN* ^25^ which expression is faint at E5 and gradually increases to be strong at the differentiation stage ^25^, ^26^ (Figure 2B). While *PDGFRA* expression is high in GI mesenchymal progenitors, its expression decreases at the onset of SMC determination and differentiation, identifying *PDGFRA* as a marker of digestive mesenchymal progenitors (Figure 2C).

### 3.3 PDGFRA expression is detected in CIPO‐SMCs

**FIGURE 3:**
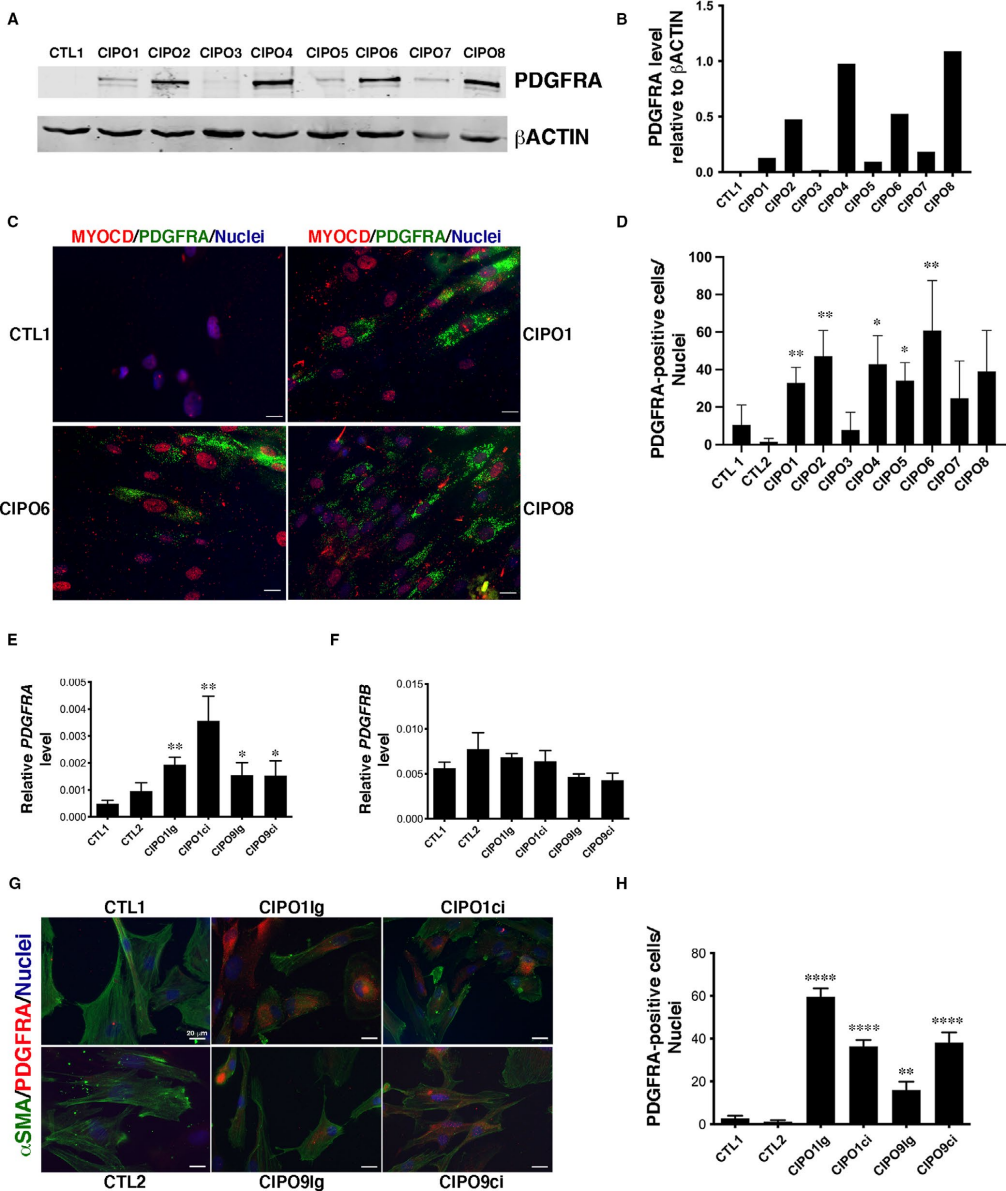
Aberrant PDGFRA expression in paediatric CIPO‐SMCs. (A) Representative Western blot analysis of PDGFRA expression in CIPO‐ and CTL‐SMC extracts. (B) Quantification relative to βACTIN expression of the Western blot results shown in A. (C) Representative immunofluorescence images of CIPO‐ and CTL‐SMC cultures incubated with anti‐PDGFRA (green) and anti‐MYOCARDIN (MYOCD, red) antibodies. Nuclei were visualized with Hoechst (blue). Scale bar = 20 µm. Percentage of PDGFRA‐positive (D) cells in CIPO‐ and CTL‐SMC cultures relative to the total number of nuclei (Hoechst staining). Values are presented as the mean ± SD of n = 5 experiments at early passages (2‐3). CTL1 and CIPO data were compared with the two‐tailed Mann‐Whitney test. RT‐qPCR analysis of *PDGFRA* (E) and *PDGFRB* (F) expression in CTL‐ and CIPO‐SMC cultures relative to *GAPDH* and *RPLPO*. Values are presented as the mean ± SD of n = 5 experiments with cells at early passages (2‐3). CTL and CIPO data were compared with the two‐tailed Mann‐Whitney test. (G) Representative immunofluorescence analysis of SMC cultures from circular and longitudinal smooth muscle layers with anti‐αSMA (green) and anti‐PDGFRA (red) antibodies. Nuclei were visualized with Hoechst (blue). Scale bar = 40 µm. Percentage of PDGFRA‐positive (H) in the indicated SMC cultures relative to the total number of cells

Our results demonstrate that SMCs of CIPO patients harbour decreased levels of contractile markers (Figure 1). As we found PDGFRA highly expressed in mesenchymal progenitors of digestive SMCs (Figure 2), we evaluated by Western blot analysis PDGFRA protein expression and found a higher level of PDGFRA protein in CIPO cultures compared to Controls (n = 7/8) (Figure 3A,B; Figure S3).

Moreover, immunofluorescence analysis of PDGFRA expression showed the elevated presence of PDGFRA‐positive cells (from 7.79% to 60.89%) in seven of the eight CIPO‐SMC cultures (Figure 3C,D) compared to Control cultures. CIPO1 SMCs were analysed by flow cytometry for surface expression of PDGFRA. Flow cytometry analysis revealed that 25.9% of CIPO1 SMCs were isolated through their PDGFRA cell surface expression (Figure S4). All PDGFRA‐positive cells in CIPO‐SMC cultures expressed MYOCARDIN in the nucleus (Figure 3C; Figures S5), indicating that PDGFRA‐positive cells derived from the SMC lineage and not from other mesenchymal cell types. Analyses of *PDGFRA* transcripts by RT‐qPCR analysis revealed a statistical increase in *PDGFRA* transcript levels in CIPO‐SMC compared to CTL‐SMC cultures both in circular and longitudinal muscle layer cultures, while *PDGFRB* levels are comparable between all conditions (Figure 3E,F). Compared to control conditions, we found that the number of PDGFRA‐positive cells was higher in CIPO‐SMC cultures both in circular and longitudinal muscle layer cultures compared to CTL‐SMC cultures (Figure 3G,H).

As digestive SMCs can redirect their cell fate upon stimulation,^45^ we next examined whether CIPO‐SMC cultures changed their fate. To this aim, we monitored by RT‐qPCR the transcript levels of *KIT*, *CD44* and *ETV1* (ICC lineage markers) and *CD34* (marker of telocytes). *CD34* was weakly expressed in Control‐SMCs. Its expression is even lower in CIPO‐SMC cultures (Figure S6). The expression level of *KIT*, *ETV1* and *CD44* was not significantly different between CIPO‐SMC and CTL‐SMC cultures (Figure S6). Similarly, the transcript levels of beta‐2 Syntrophin (*SNTB2*), which is expressed in the human circular and longitudinal smooth muscle layers, independently of their differentiation status,^46^ were comparable in CIPO‐ and CTL‐SMC cultures (Figure S6), strongly arguing in favour of a SMC identity for CIPO cells. The status of PDGFRA‐positive cells was examined at early (2‐3) and late (7‐8) passages. We found that the percentage of PDGFRA‐positive cells remained constant in CIPO‐SMC, and that the PDGFRA expression was not induced in CTL‐SMC cultures (Figure S7A). In contrast, the proliferative index of SMC cultures detected by Ki67 immunostaining was reduced at late passages in almost all cultures (Figure S7B) suggesting a decrease in proliferative capacity after more than 3 passages. We switched from a serum‐enriched medium to a medium supplemented with BSA and insulin to stimulate SMC differentiation.^24^, ^28^ While this stimulation led to a better organization of αSMA‐labelling in all SMC cultures (compare Figure S8 and Figure 1A), it did neither affect the aberrant expression of PDGFRA nor the low expression of αSMA in CIPO‐SMC cultures (Figure S8). Thus, our findings reveal a decreased expression of SMC contractile markers in CIPO‐SMC. This phenotype is accompanied by an increase in the expression of PDGFRA, a gene expressed during embryonic gut development that defines mesenchymal progenitors. These results suggest a phenotypic switch of the CIPO‐SMCs towards an undifferentiated stage. This modulation occurs in CIPO‐SMC without origin‐related differences as comparable observations were done both in longitudinal and circular smooth muscle layers.

### 3.4 A functional PDGFRA signalling pathway is present in human CIPO‐SMC cultures

**FIGURE 4:**
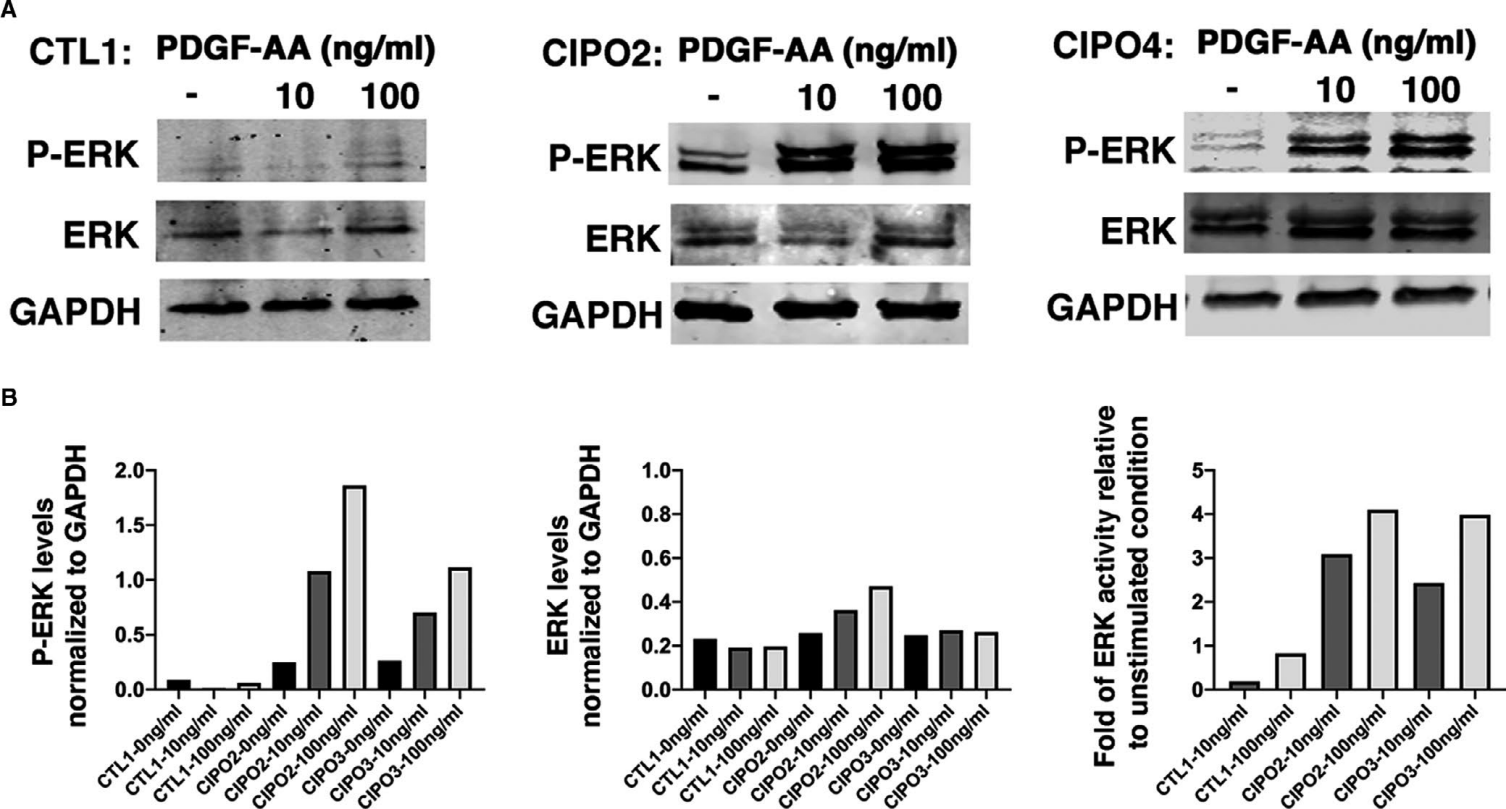
PDGFRA is functional in CIPO‐SMCs. (A) Representative immunoblots showing phosphorylated ERK (p‐ERK) and total ERK in CIPO2‐, CIPO4‐ and CTL1‐SMC culture that were serum starved before stimulation or not (‐) with the indicated concentrations of PDGF‐AA. Protein loading was verified with an anti‐GAPDH antibody. Quantification of the Western blot data in (B). P‐ERK and ERK levels were normalized to GAPDH expression (left and middle panels). Normalized expression levels were converted to fold changes compared with CTL without stimulation (set to 1) for ERK activity (right panel)

We next assessed PDGFRA signalling pathway activity in CIPO‐SMCs. We noticed that the basal ERK signalling activity, monitored through the phosphorylation of ERK (p‐ERK), was higher in CIPO‐SMCs compared to control cultures (Figure 4A,B). Furthermore, stimulation of CIPO‐SMCs with increasing doses of PDGF‐AA, the specific ligand of this receptor, for 10 minutes after serum deprivation, induced an increase in the endogenous PDGFRA activity in CIPO‐SMCs. In Control‐SMCs, P‐ERK remained unchanged upon this stimulation (Figure 4A,B). All together, these data demonstrate that the expression of PDGFRA in CIPO‐SMCs is associated with a higher basal level of ERK activity and to a higher capacity of CIPO cells to respond to PDGFRA.

### 3.5 The phenotypic switch is present in intestinal muscle tissues from paediatric CIPO

**FIGURE 5:**
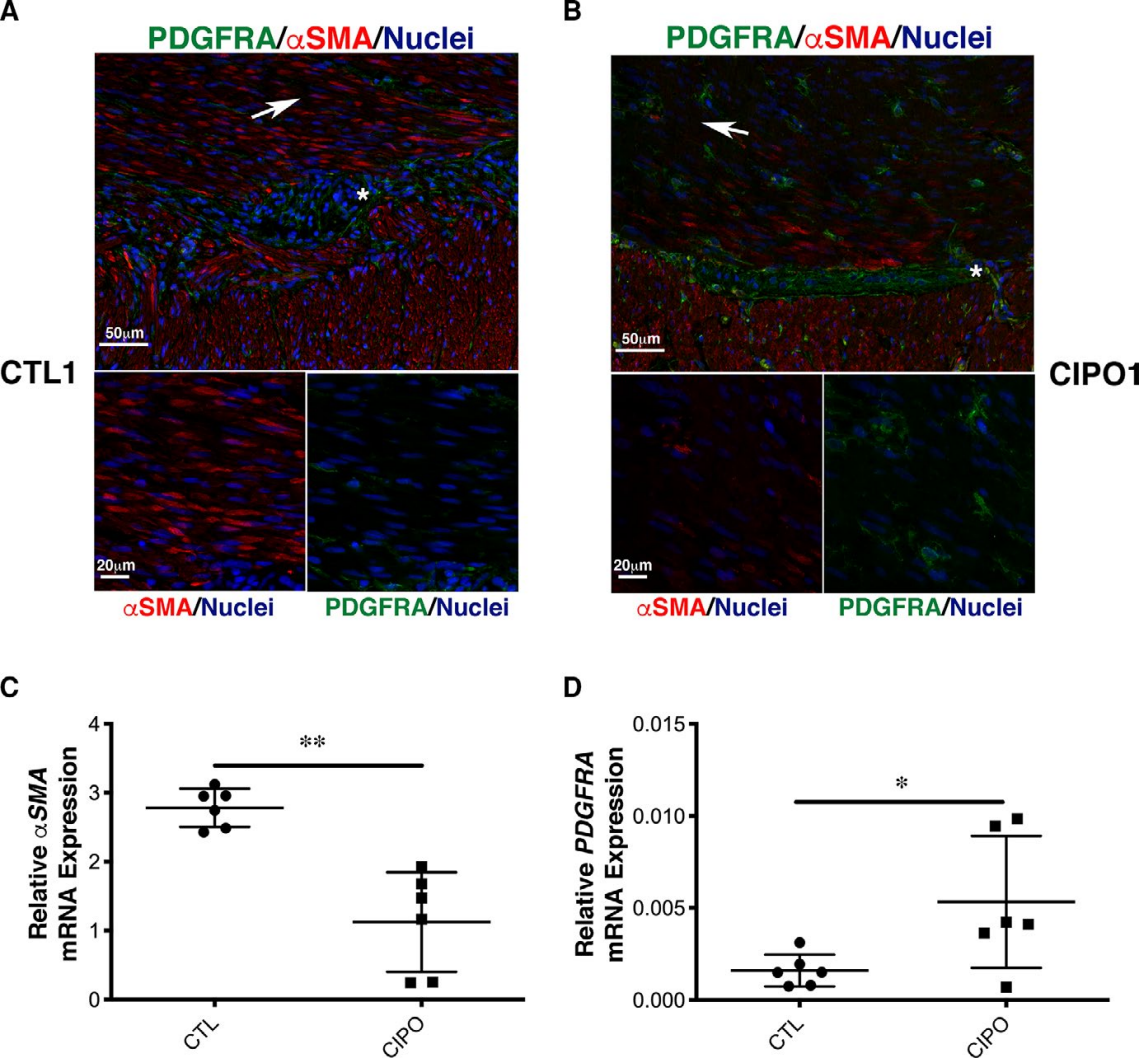
Immunodetection of PDGFRA and αSMA in full‐thickness intestinal biopsies from children with CIPO syndrome. All biopsies are oriented to show the circular muscle layer on top and the longitudinal layer at the bottom. Tissue sections from (A) the normal ganglionic zone in a control biopsy from a patient with Hirschsprung's disease patient (CTL1) and (B) from a biopsy of a child with CIPO syndrome (CIPO1) were incubated with rabbit anti‐PDGFRA and mouse anti‐αSMA antibodies. Nuclei were visualized with Hoechst (blue). Scale bars = 50 µm and 20 µm for enlargement. Images are representative of triplicate experiment. White arrows indicate the below enlargement. White asterisk indicates myenteric plexus. RT‐qPCR analysis of (C) α*SMA* expression and (D) *PDGFRA* expression in CIPO (n = 6) and CTL (n = 6) intestinal muscle layers; values (mean ± SD) are relative to *GAPDH* and *RPLPO* mRNA expression

To evaluate the differentiation status of the SMC in paediatric CIPO tissues, we examined αSMA and PDGFRA expression by immunofluorescence. In control tissues (normal zone of intestinal specimens from children with Hirschsprung's disease), αSMA expression was found localized both in the circular and longitudinal smooth muscle layers (Figure 5A). We detected a faint expression of PDGFRA mainly localized in cells located around the myenteric plexus. Additional staining was found in the circular smooth muscle layer in cells that could correspond to telocytes, as previously observed in human and murine digestive tissues ^41^, ^47^, ^48^ (Figure 5A; Figure S9). In contrast, in CIPO samples, αSMA expression was strongly reduced compared to controls, especially in the circular layer (Figure 5B; Figure S9). Conversely, the number of PDGFRA‐positive cells located in the circular and longitudinal smooth muscle layers was substantially increased (Figure 5B; Figure S9). We also observed PDGFRA expression in the myenteric plexus in some CIPO patients (Figure 5B). We quantified these observations by performing by RT‐qPCR on six CIPO and six control intestinal muscle fibre samples. Compared to controls, *αSMA* mRNA level was reduced (n = 6/6) (Figure 5C) and *PDGFRA* mRNA level was higher (n = 5/6) (Figure 5D) in CIPO specimens. As observed in cultures, we found that the SMCs of paediatric CIPO specimens analysed harbour a phenotypic switch towards an undifferentiated stage.

## 4 DISCUSSION

Recent genetic studies on the CIPO syndrome identified mutations in multiple genes involved in smooth muscle function and structure, supporting the hypothesis that smooth muscle alterations contribute to the CIPO phenotype. Currently, there are few mouse models with specific gene mutation that specifically affected smooth muscle in the colon or small intestine,^24^, ^49^, ^50^ but no polymorphism has been identified so far in CIPO patients. Digestive SMCs can dedifferentiate upon stimulation and the anarchic control and reactivation of this mechanism could be implicated in digestive motility disorders. Previous works demonstrated that in adults, SMC cultures could lead to the identification of aberrant molecular mechanisms involved in digestive pathologies, particularly inflammatory bowel disease and Crohn's disease.^3^, ^4^ However, no study focused on paediatric gastrointestinal diseases such as the CIPO syndrome.

In this study, we set up a culture model of paediatric CIPO syndrome. We isolated cells from intestinal smooth muscle fibres of paediatric patients with CIPO and found strong and homogeneous expression of MYOCARDIN in CIPO cultures demonstrating that CIPO cells derived from patient tissues are homogenous and from the SMC lineage. The GI smooth muscle is a complex structure composed of multiple mesenchyme‐derived cells such as SMCs, ICCs, telocytes and sub‐epithelial myofibroblasts.^2^, ^41^, ^51^ Transplasticity has been described in the GI tract, and during childhood, KIT activity blockage induces ICC trans‐differentiation into SMCs.^45^ Our experiment clearly demonstrated that CIPO‐SMC cultures in our conditions maintain the smooth muscle identity, as demonstrated by the nuclear expression of the MYOCARDIN, and did not switch to an ICC‐ or telocyte‐derived cells. To our knowledge, it is the first study reporting a successful culture model of paediatric CIPO syndrome.

We also reveal that despite the expression of MYOCARDIN, CIPO‐SMCs harbour low level of αSMA compared to control cells independently of the origin of the smooth muscle (circular or longitudinal). Alterations in smooth muscle contractile proteins were described in case reports of paediatric and adult CIPO ^4^, ^12^, ^13^ and were interpreted as a dysmotility marker.^6^ Indeed, difference in αSMA expression in the small and large intestine smooth muscle has been a source of debate.^19^ Here, we showed that αSMA protein level is reduced in CIPO‐SMC cultures and in smooth muscle samples from children with CIPO, suggesting that paediatric CIPO aetiology is associated in part to smooth muscle dedifferentiation.

**FIGURE 6:**
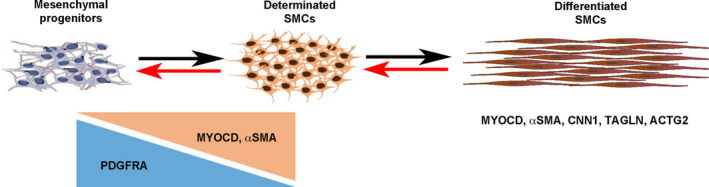
Model of the gastrointestinal SMC development and plasticity versus PDGFRA expression

Our long‐term studies on the development of the GI tract have identified signalling pathways that regulate digestive mesenchymal progenitor proliferation. Such signalling pathways could be deregulated in pathological conditions, leading to the dedifferentiation of functional SMCs.^24^, ^26^, ^28^ In this study, we demonstrated that PDGRFA expression is strongly observed in the whole GI tract mesenchyme and its dynamics characterizes the digestive mesenchymal progenitors in chick embryos (Figure 6). PDGFR pathways have been involved in the development and differentiation of many organs.^43^ PDGFRs have been established as key survival factors for vascular SMCs, and principal regulators of vascular SMC phenotype.^44^ PDGFRB plays a critical role in the migration of vascular SMCs,^52^ whereas no involvement of PDGFRA was found.^44^ PDGFRA‐expressing cells are also found in gut, lung and kidney mesenchyme.^53^ Extensive data indicate that PDGFRA‐positive cells located close to sub‐epithelial layer regulate epithelial homeostasis.^54^ PDGFRA‐positive cells that characterize the presence of telocytes, an interstitial cell type, are present into the whole GI tract and are organized to form 3‐D networks in the submucosa and in the interstitium between the longitudinal and circular muscle layers.^41^, ^42^ During the development, PDGFRA is dynamically expressed in the common mesenchymal progenitors of ICC and SMC ^47^ in the developing and undifferentiated mouse intestinal mesenchyme. Moreover, selective PDGFR inhibition suppresses intestinal SMC differentiation.^47^ However, no study investigated the expression of PDGFR family members in paediatric intestinal motility disorders. We found that PDGFRA expression was elevated in intestinal smooth muscle samples from patients with CIPO and also in CIPO‐SMCs that we derived in cultures, where *PDGFRB* expression was not affected. Our flow cytometry and Western blot analyses reveal that a PDGFRA/ERK signalling is active in CIPO‐SMCs (Figure 6). Recently, it was reported that the number of PDGFRA‐positive cells is increased in hypertrophic intestinal smooth muscle in mice that underwent partial obstruction surgery,^55^ but this was not correlated with MYOCARDIN expression. As PDGFRA has been shown to be expressed in the telocytes in normal small intestine, one could imagine an increase in the number of telocytes in CIPO‐SMCs. However, we found that all PDGFRA‐positive cells in CIPO culture also expressed MYOCARDIN in the nuclear compartment, defining these cells as smooth muscle‐derived cells.

In conclusion, our data strongly suggest that a phenotypic switch occurs in the SMCs of paediatric CIPO patients and highlight the PDGFRA signalling pathway as a target for drug design for CIPO syndrome.

## CONFLICT OF INTEREST

The authors confirm that there are no conflicts of interest.

## AUTHOR CONTRIBUTION

**Delphine Martire:** Formal analysis (equal); Investigation (equal). **Sarah Garnier:** Formal analysis (equal); Investigation (equal). **Sébastien Sagnol:** Investigation (equal). **Annick Bourret:** Investigation (equal). **Stéphane Marchal:** Methodology (equal). **Norbert Chauvet:** Methodology (equal). **Amandine Guérin:** Investigation (equal). **Dominique Forgues:** Resources (equal). **dominique Berrebi:** Resources (equal). **Christophe Chardot:** Resources (equal). **Marc Bellaiche:** Resources (equal); Validation (equal). **John Rendu:** Investigation (equal); Resources (equal). **Nicolas Kalfa:** Resources (equal). **Sandrine Faure:** Methodology (equal); Validation (equal); Writing‐review & editing (equal). **Pascal de Santa Barbara:** Supervision (equal); Validation (equal); Writing‐original draft (equal); Writing‐review & editing (equal).

## Supporting information

Supplementary Material

## Data Availability

The data that support the findings of this study are available from the corresponding author upon request.
