# Supplementary material for: Phenotypic switch of smooth muscle cells in paediatric chronic intestinal pseudo‐obstruction syndrome
Source: J Cell Mol Med. 2021 Mar 3;25(8):4028–39. doi: 10.1111/jcmm.16367 (PMC8051695; doi:10.1111/jcmm.16367)
Supplement: Supplementary file 1 — Supplementary Material [file JCMM-25-4028-s001.pdf]

Supplementary Table and Figures

Table. S1 Sequences of the primers used for RT-qPCR.

Figure S1 Immunofluorescence of αSMA (smooth muscle marker, red) and TUJ1 (neuronal marker, green) expression in the ganglionic zone of the biopsies from two children with Hirschsprung's disease (used as controls in this study). Circular (circul.) and longitudinal (longit.) smooth muscle layers are indicated. Nuclei were visualized with Hoechst (blue). Scale bar = 50 μm.

Figure S2 Sanger sequencing confirmation of the c.588G>C (p.E196D) variant identified in patient CIPO1. Chromatograms shows the mutant sequence (top) and the reference. The heterozygous variant is indicated by the arrow.

Figure S3 Representative western blot analysis of αSMA and PDGFRA expression in CIPO- and CTL-SMC extracts (**A**). (**B**) Quantification relative to GAPDH expression of the western blot results shown in **A**.

Figure S4 Membrane expression of PDGFRA in CIPO SMC culture. CIPO1 SMCs were dissociated and analyzed in the presence of PE-mouse anti-human CD140a antibodies (**A**) or with PE alone (**B**) in flow cytometry to isolate and to quantify PDGFRA-loacted at the cell surface.

Figure S5 Representative immunofluorescence analysis of the indicated SMC cultures with anti-PDGFRA (green) and anti-MYOCD (red) antibodies (**A**). Nuclei were visualized with Hoechst (blue). Scale bar = 20 μm. (**B**) Table represents the total number of SMCs (Hoescht staining), MYOCARDIN- and PDGFRA-expressing SMCs of n=4 experiments with cells at early passages (2-3). Relative PDGFRA-positive/MYOCD-positive and MYOCD-positive/ PDGFRA-positive SMCs were quantified for each conditions.

Figure S6 Confirmation of SMC identity in CTL and CIPO-SMC cultures. RT-qPCR analysis of *KIT*, *ETV1*, *CD44*, *SNTB2*, and *CD34* in SMC cultures relative to *GAPDH* and *RPLPO*. All values are presented as the mean ± SD of n=5 experiments with cells at different passages. CIPO and CTL data were compared with the two-tailed Mann-Whitney test.

Figure S7 PDGFRA expression in CIPO- and CTL-SMC cultures at early (number) and late passage (number). Number of PDGFRA-expressing SMCs (**A**) and KI67-expressing SMCs (**B**) relative to the total number of cells at early and late passage. Values are the mean ± SD of n=5 experiments. Early and late passage values were compared with the two-tailed Mann-Whitney test.

Figure S8 Analysis of human CIPO SMC cultures incubated with BSA/insulin and without FBS for 6 days. (**A**) Representative immunofluorescence images of SMC cultures incubated with anti-αSMA (green) and anti-MYOCD (red) antibodies. Nuclei were visualized with Hoechst (blue). Scale bar = 20 μm. (**B**) Representative immunofluorescence images of SMC cultures incubate d with anti-αSMA (green)

and anti-PDGFRA (red) antibodies. Nuclei were visualized with Hoechst (blue). Scale bar = 20 μm. Percentage of (**C**) MYOCARDIN-, (**D**) αSMA- and (**E**) PDGFRA-expressing SMCs in the indicated SMC cultures relative to the total number of cells. (**C-E**) Values are presented as the mean ± SD of n=3 experiments during early passages. CTL1 and CIPO data were compared with the two-tailed Mann-Whitney test.

Figure S9 Immunofluorescence of PDGFRA in full-thickness intestinal biopsies from children with CIPO syndrome and from controls (CTL). All biopsies are oriented to show the circular smooth muscle layer on top and the longitudinal smooth muscle layer at the bottom. Tissue sections from (**A**) the normal zone of biopsies from children with Hirschsprung's disease (CTL2 and CTL3) and from (**B**) biopsies of children with CIPO (CIPO8 and CIPO9) were probed with rabbit anti-PDGFRA antibodies. Nuclei were visualized with Hoechst (blue). Scale bar = 50 μm. White arrowheads: circular smooth muscle layer; white arrows: longitudinal smooth muscle layer; red arrowheads: myenteric plexus.

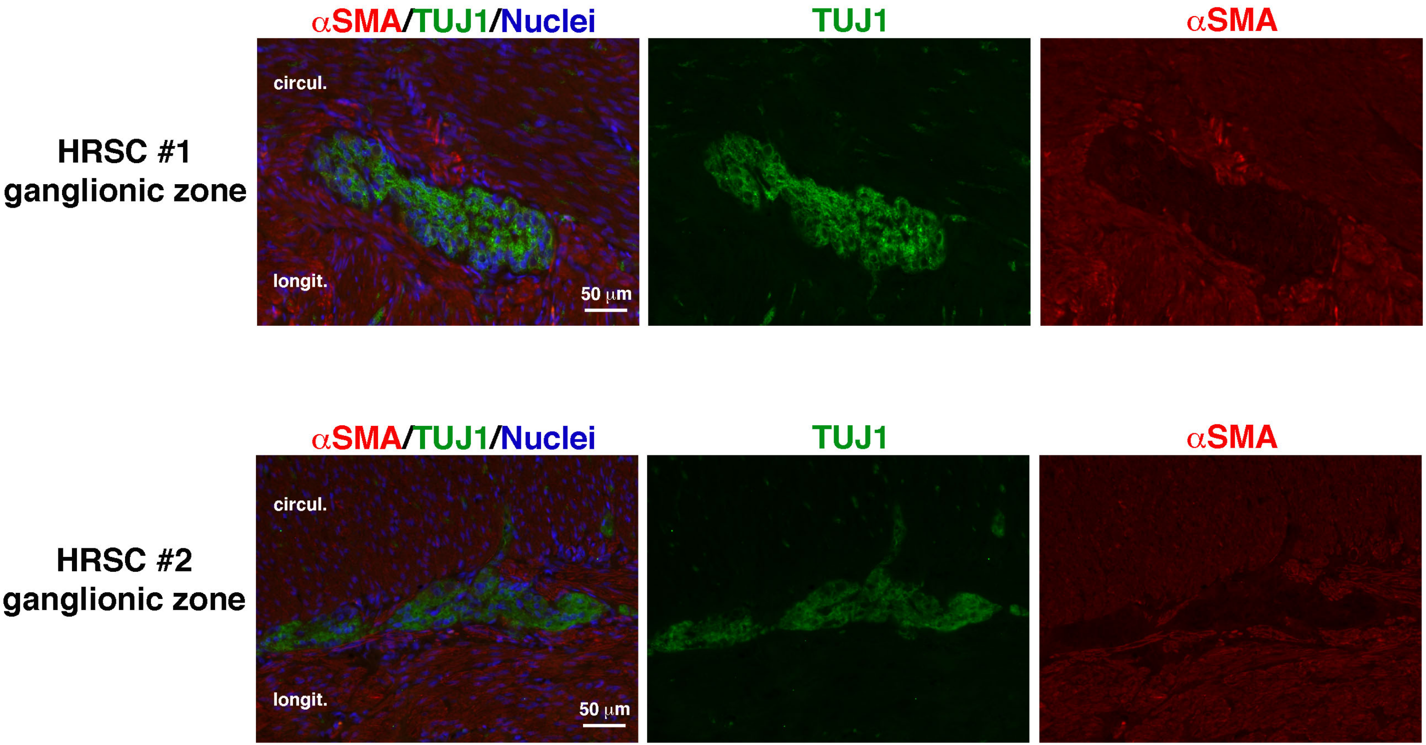
αSMA/TUJ1/Nuclei
TUJ1
αSMA
HRSC #1
ganglionic zone
circul.
longit.
50 μm
αSMA/TUJ1/Nuclei
TUJ1
αSMA
HRSC #2
ganglionic zone
circul.
longit.
50 μm

Figure S1

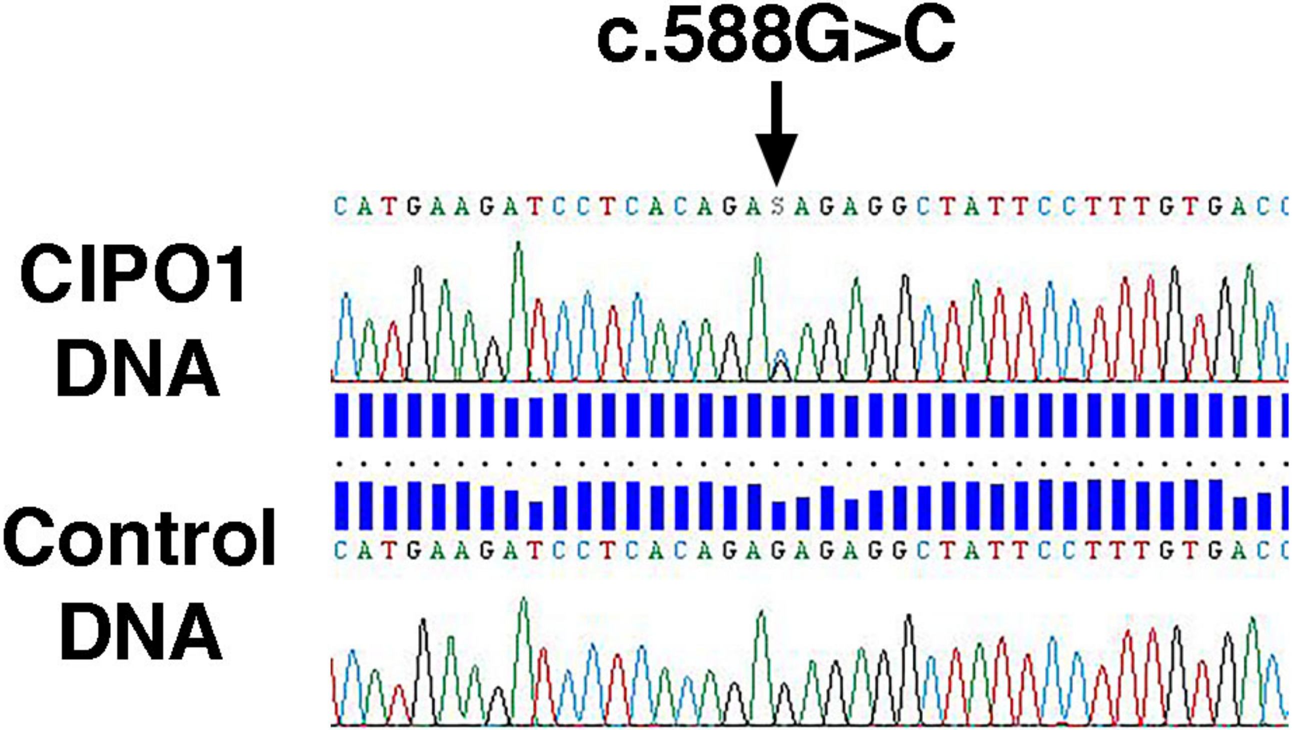
c.588G>C
CATGAAGATCCTCACAGASAGAGGCTATTCCTTTGTGAC
CIPO1 DNA
Control DNA
CATGAAGATCCTCACAGAGAGAGGCTATTCCTTTGTGAC
DNA

Figure S2

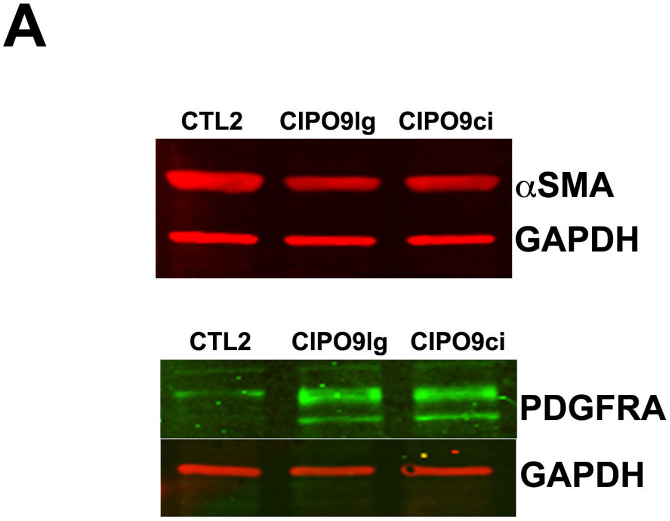
A
CTL2
CIPO9lg
CIPO9ci
αSMA
GAPDH
CTL2
CIPO9lg
CIPO9ci
PDGFRA
GAPDH

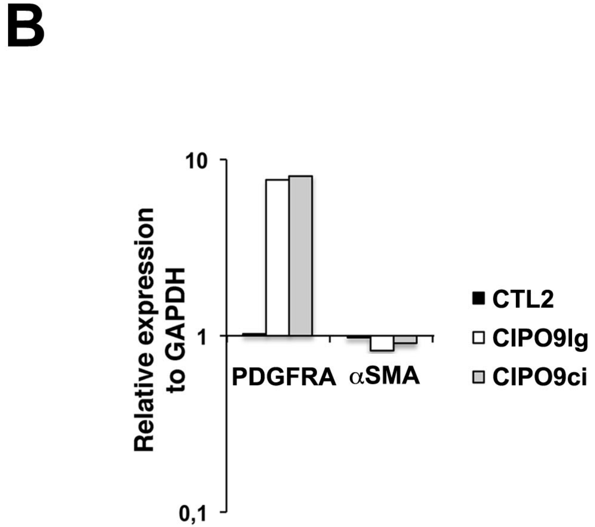
B
Relative expression to GAPDH
10
1
0,1
PDGFRA
αSMA
CTL2
CIPO9lg
CIPO9ci

Figure S3

A

CIPO1

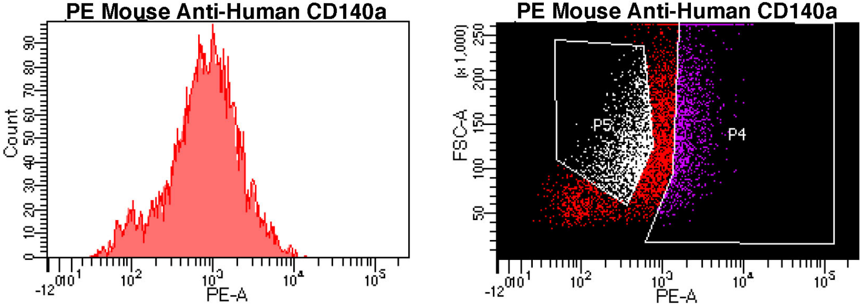

Population	#Events	%Parent	%Total
All Events	10,000	####	100.0
P1	8,037	80.4	80.4
P2	6,087	75.7	60.9
P3	4,852	79.7	48.5
→ P4	1,257	25.9	12.6
P5	1,466	30.2	14.7

→ 25.9% of PDGFRA+ cells

B

CIPO1 Neg. Control

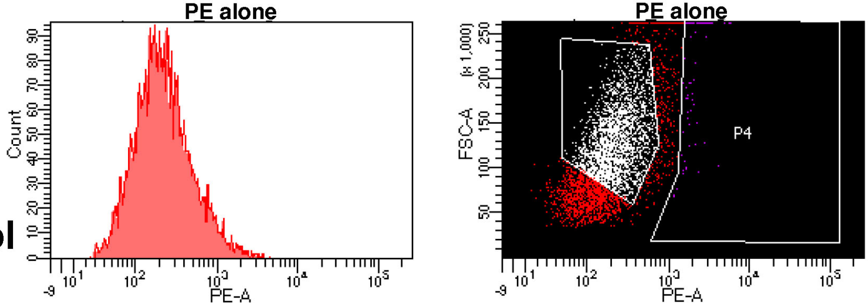

Population	#Events	%Parent	%Total
All Events	10,000	####	100.0
P1	5,890	58.9	58.9
P2	4,664	79.2	46.6
P3	3,927	84.2	39.3
→ P4	79	2.0	0.8
P5	2,353	59.9	23.5

→ 2% of PDGFRA+ cells

Figure S4

A

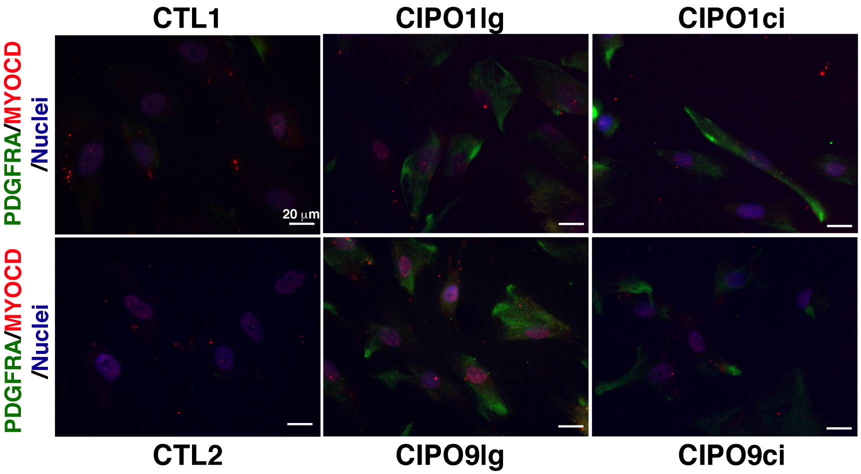

B

	CTL1	CTL2	CIPO1lg	CIPO1ci	CIPO9lg	CIPO9ci
number of SMCs (DAPI)	308	367	243	252	403	331
number of MYOCD-positive SMCs	308	366	239	251	397	331
number of PDGFRA-positive SMCs	21	5	207	147	136	66
relative PDGFRA-positive/MYOCD-positive SMCs	6.81%	1.3%	85.18%	58.33%	33.74%	19.93%
relative MYOCD-positive/PDGFRA-positive SMCs	100%	100%	98.35%	99.6%	98.5%	100%

Figure S5

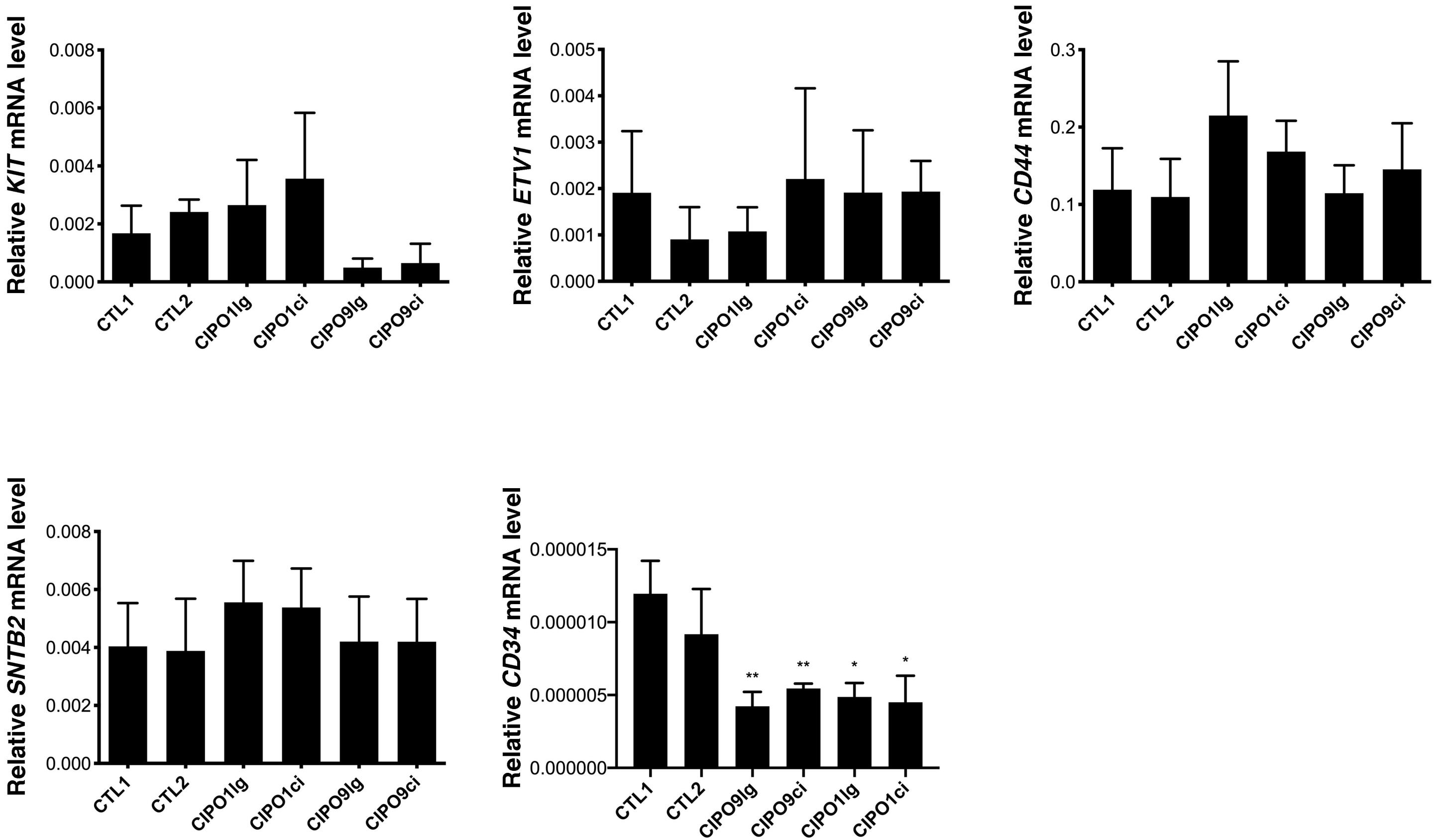
Relative KIT mRNA level
0.008
0.006
0.004
0.002
0.000
CTL1
CTL2
CIPO1lg
CIPO1ci
CIPO9lg
CIPO9ci
Relative ETV1 mRNA level
0.005
0.004
0.003
0.002
0.001
0.000
CTL1
CTL2
CIPO1lg
CIPO1ci
CIPO9lg
CIPO9ci
Relative CD44 mRNA level
0.3
0.2
0.1
0.0
CTL1
CTL2
CIPO1lg
CIPO1ci
CIPO9lg
CIPO9ci
Relative SNTB2 mRNA level
0.008
0.006
0.004
0.002
0.000
CTL1
CTL2
CIPO1lg
CIPO1ci
CIPO9lg
CIPO9ci
Relative CD34 mRNA level
0.000015
0.000010
0.000005
0.000000
**
**
*
*
CTL1
CTL2
CIPO9lg
CIPO9ci
CIPO1lg
CIPO1ci

Figure S6

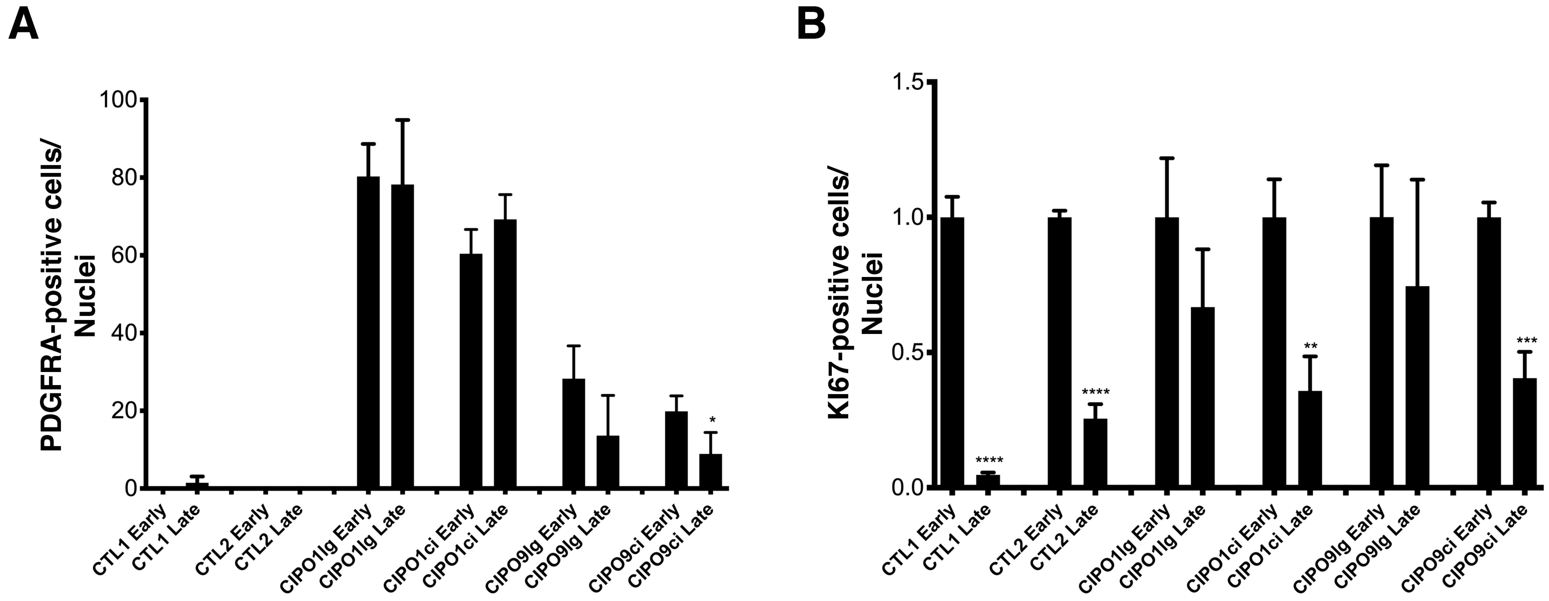
A
PDGFRA-positive cells/
Nuclei
100
80
60
40
20
0
*
CTL1 Early
CTL1 Late
CTL2 Early
CTL2 Late
CIPO1lg Early
CIPO1lg Late
CIPO1ci Early
CIPO1ci Late
CIPO9lg Early
CIPO9lg Late
CIPO9ci Early
CIPO9ci Late
B
KI67-positive cells/
Nuclei
1.5
1.0
0.5
0.0
****
****
**
***
CTL1 Early
CTL1 Late
CTL2 Early
CTL2 Late
CIPO1lg Early
CIPO1lg Late
CIPO1ci Early
CIPO1ci Late
CIPO9lg Early
CIPO9lg Late
CIPO9ci Early
CIPO9ci Late

Figure S7

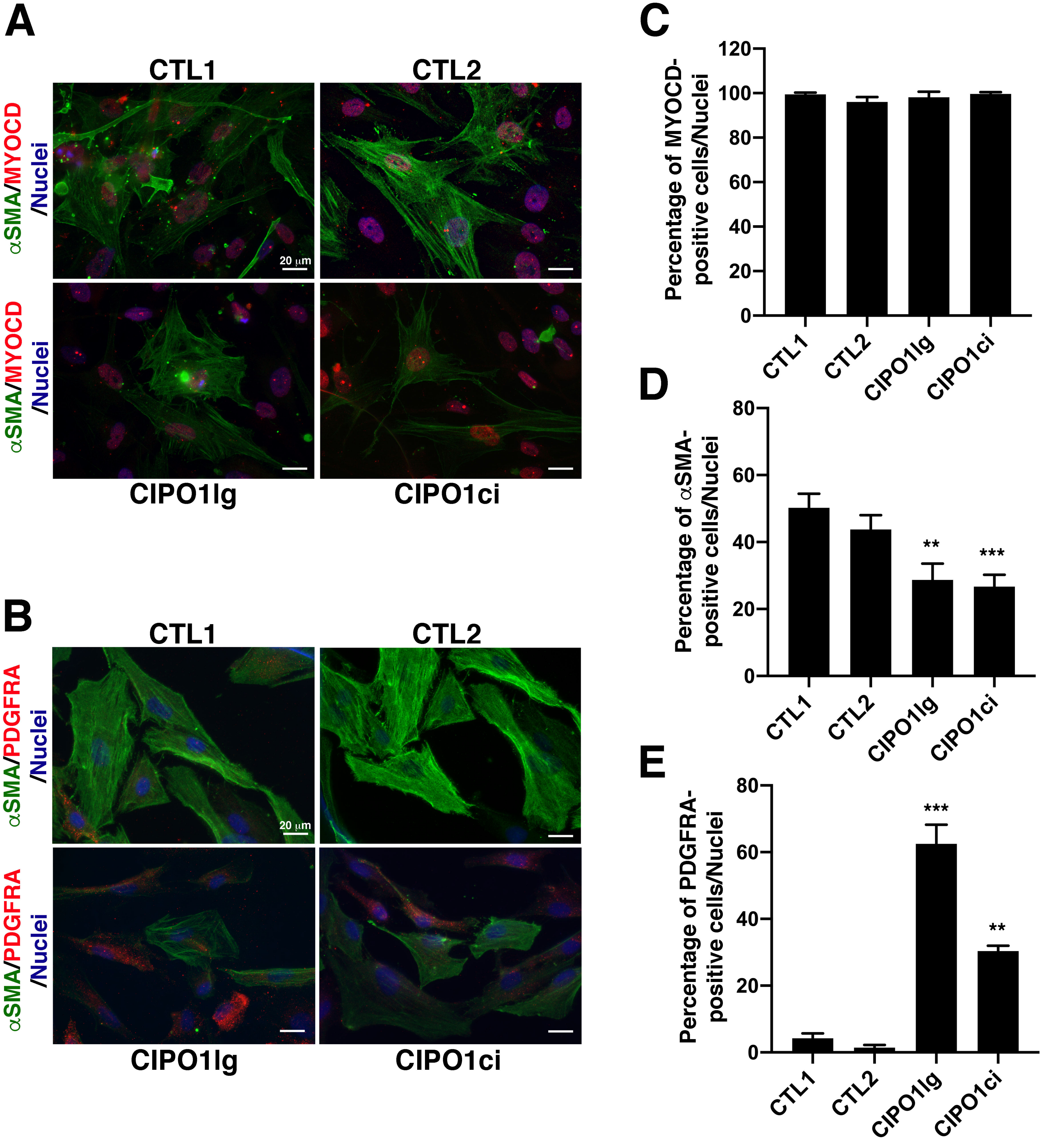
A
CTL1
CTL2
αSMA/MYOCD/Nuclei
20 μm
CIPO1lg
CIPO1ci
B
CTL1
CTL2
αSMA/PDGFRA/Nuclei
20 μm
CIPO1lg
CIPO1ci
C
Percentage of MYOCD-positive cells/Nuclei
120
100
80
60
40
20
0
CTL1
CTL2
CIPO1lg
CIPO1ci
D
Percentage of αSMA-positive cells/Nuclei
80
60
40
20
0
**
***
CTL1
CTL2
CIPO1lg
CIPO1ci
E
Percentage of PDGFRA-positive cells/Nuclei
80
60
40
20
0
***
**
CTL1
CTL2
CIPO1lg
CIPO1ci

Figure S8

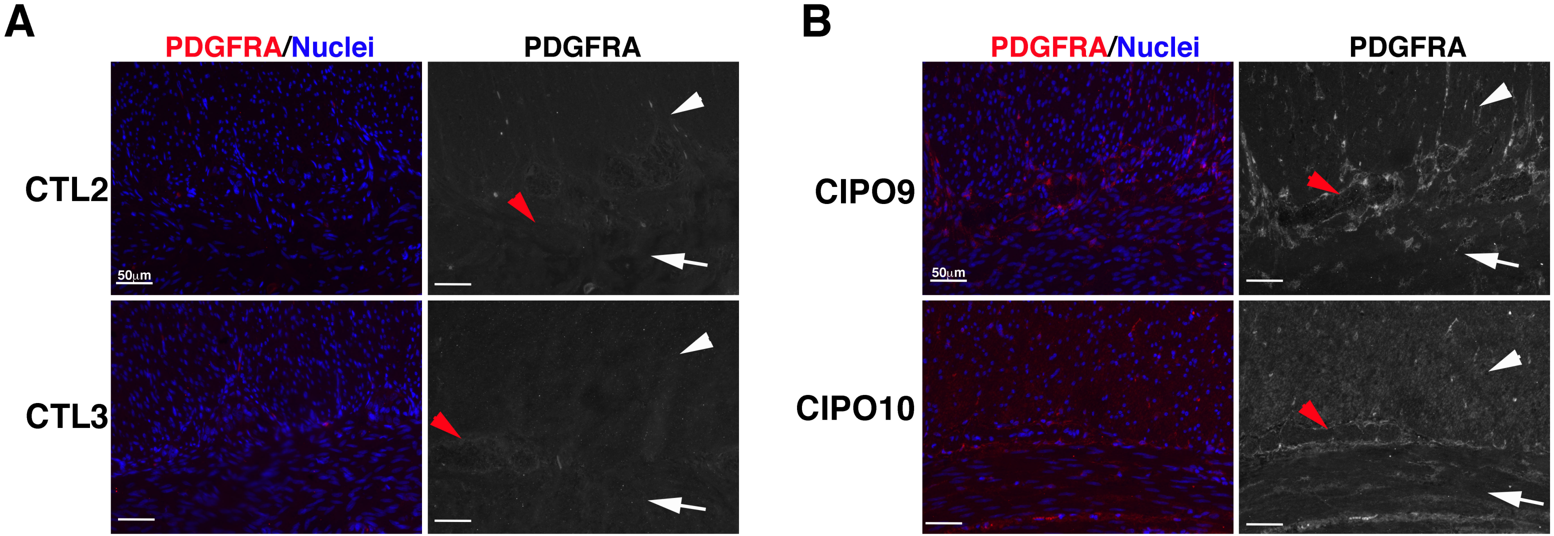
A
PDGFRA/Nuclei
PDGFRA
CTL2
CTL3
50μm
B
PDGFRA/Nuclei
PDGFRA
CIPO9
CIPO10
50μm

Figure S9

Supplemental Table 1: Gene-specific human primers used for RT-qPCR.

Targets	Forward primer (5'-3')	Reverse primer (5'-3')	Amplicon (bp)
CD34	TAG CCT GTC ACC TGG AAA TG	GTC ACT TAG GAT AGG AGA AGA TGA T	103
CD44	TGA TGG CAC CCG CTA TG	CTT TCA CTG GAG GAG CCG	108
ETV1	CAC CTG TGT TGT CCC AGA AA	CTG CCA GAG CTG AAG TGA	106
GAPDH	CCA TCT TCC AGG AGC GAG	CTT GAG GCT GTT GTC ATA CT	212
KIT1	CCT TTG CTG ATT GGT TTC G	AGG AAG TTG TGT TGG GTC TA	143
PDGFRA	ATG TGC CAG ACC CAG AT	CCC TCA CTG TTG TGT AAG GTT	139
PDGFRB	TGG TAT CTT TGA GGA CAG AAG C	ATG GAT GAC ACC TGG AGT	102
RPLPO	TCA TCC AGC AGG TGT TCG	AGC AAG TGG GAA GGT GTA A	206
SNTB2	TCA AGT TCA TCC GAG AAG TAA CAC	AGA GTC CTC ACT GCC ACT AAA	113
